# Physical activity and sedentary time during pregnancy and associations with maternal and fetal health outcomes: an epidemiological study

**DOI:** 10.1186/s12884-021-03627-6

**Published:** 2021-02-27

**Authors:** Lina Meander, Maria Lindqvist, Ingrid Mogren, Jonas Sandlund, Christina E. West, Magnus Domellöf

**Affiliations:** 1 Department of Clinical Sciences, Pediatrics, Umeå University, Umeå, Sweden; 2 Department of Nursing, Umeå University, Umeå, Sweden; 3 Department of Clinical Sciences, Obstetrics and Gynecology, Umeå University, Umeå, Sweden; 4 Department of Community Medicine and Rehabilitation, Umeå University, Umeå, Sweden

**Keywords:** Physical activity, Sedentary time, Pregnancy outcomes, Gestational weight gain, Epidemiological study

## Abstract

**Background:**

Physical activity is generally considered safe for the pregnant woman as well as for her fetus. In Sweden, pregnant women without contraindications are recommended to engage in physical activity for at least 30 min per day most days of the week. Physical activity during pregnancy has been associated with decreased risks of adverse health outcomes for the pregnant woman and her offspring. However, there are at present no recommendations regarding sedentary behavior during pregnancy. The aim was to examine the level of physical activity and sedentary time in a representative sample of the pregnant population in Sweden, and to explore potential effects on gestational age, gestational weight gain, birth weight of the child, mode of delivery, blood loss during delivery/postpartum, self-rated health during pregnancy and risk of pregnancy-induced hypertension and preeclampsia.

**Methods:**

This was an epidemiological study using data from the prospective, population-based NorthPop study in Northern Sweden and information on pregnancy outcomes from the national Swedish Pregnancy Register (SPR). A questionnaire regarding physical activity and sedentary time during pregnancy was answered by 2203 pregnant women. Possible differences between categories were analyzed using one-way Analysis of variance and Pearson’s Chi-square test. Associations between the level of physical activity/sedentary time and outcome variables were analyzed with univariable and multivariable logistic regression and linear regression.

**Results:**

Only 27.3% of the included participants reported that they reached the recommended level of physical activity. A higher level of physical activity was associated with a reduced risk of emergency caesarean section, lower gestational weight gain, more favorable self-rated health during pregnancy, and a decreased risk of exceeding the Institute of Medicine’s recommendations regarding gestational weight gain. Higher sedentary time was associated with a non-favorable self-rated health during pregnancy.

**Conclusions:**

Our study showed that only a minority of pregnant women achieved the recommended level of physical activity, and that higher physical activity and lower sedentary time were associated with improved health outcomes. Encouraging pregnant women to increase their physical activity and decrease their sedentary time, may be important factors to improve maternal and fetal/child health outcomes.

**Supplementary Information:**

The online version contains supplementary material available at 10.1186/s12884-021-03627-6.

## Background

Physical activity (PA) is associated with reduced mortality and reduced risk of several major noncommunicable diseases such as cardiovascular disease, hypertension and type 2 diabetes in the general population [1]. The World Health Organization (WHO) recommends that all individuals between 18 and 64 years of age should perform moderate intensity PA of at least 150 min per week, or 75 min of high intensity PA per week, or a combination of these [2]. Moderate intensity PA during pregnancy is considered safe for both the pregnant woman and her fetus, and in Sweden pregnant women without medical contraindications are encouraged to engage in PA of at least 30 min per day most days of the week [3]. In addition to the benefits of PA in general, PA during pregnancy is associated with decreased risk of gestational diabetes mellitus, preeclampsia, pregnancy-induced hypertension and excessive gestational weight gain (GWG) [4, 5]. PA during pregnancy is also associated with improved psychological well-being and reduced risk of postpartum depressive symptoms [6]. Nevertheless, previous studies show that a large proportion of pregnant women do not reach the recommendations, and time allocated to PA tends to decrease during the course of pregnancy [7].

Even for an individual who reaches the recommended level of PA, it is still common to spend excessive time engaging in sedentary behavior [7]. Sedentary behavior is defined as activities that include energy expenditure between 1.0 and 1.5 metabolic equivalent units (METs), like sitting, sleeping and watching television [8]. Sedentary behavior is related to all-cause mortality, cardiovascular disease and type 2 diabetes in adults, regardless of level of PA [9]. Studies show that women tend to increase their sedentary time when they become pregnant. The effect of sedentary time is not as well investigated as the effect of PA on pregnancy outcomes, and the possible associations between sedentary time and GWG, hypertensive disorders during pregnancy, and birth weight still remain uncertain [10].

Pregnancy-induced hypertension is defined as blood pressure 140/90 mmHg or more that is diagnosed after 20 weeks of gestation in previously normotensive women. Preeclampsia is defined as pregnancy-induced hypertension accompanied by new onset of at least one of the following conditions after 20 weeks of gestation: proteinuria, maternal acute kidney injury, liver dysfunction, neurological symptoms, hematological complications or uteroplacental dysfunction like fetal growth restriction [11]. Hypertensive disorders affect about 8% of pregnant women and are major causes of maternal and fetal morbidity [12]. Excessive postpartum blood loss is another cause of maternal morbidity and there is an increasing incidence of women diagnosed with postpartum hemorrhage [13]. Finding modifiable risk factors for these disorders are therefore important for the health of the pregnant women.

High body mass index (BMI) and excessive GWG are increasing problems in many countries, and almost 50% of pregnant women in the United States and Europe gain more weight than recommended. This results in an increased risk of the offspring being large for gestational age, and thus exposed to an increased risk of caesarean section (CS) [14]. High birth weight and excess GWG are also associated with increased risk of childhood obesity [15]. Clarifying the effect of PA and sedentary time on these outcomes may motivate the pregnant women to a healthy lifestyle throughout pregnancy.

Previous studies performed in Sweden have focused on investigation of the pre-pregnant PA level and PA until gestational age 10 weeks in relation to pregnancy outcomes [16], while this study aimed to evaluate the level of PA and sedentary time in pregnancy up to gestational age 32–34 weeks, and further evaluate the impact on maternal and fetal/child health outcomes during pregnancy and delivery.

## Methods

### Study design and participants

This was an epidemiological study using data from the prospective, population-based NorthPop study in Northern Sweden and the national SPR. All eligible pregnant women in the Umeå University Hospital catchment area during the study period were invited to participate in the study at the time of their routine ultrasound examination around gestational age 17–20 weeks. The inclusion criteria were as follows: pregnant woman ≥18 years of age, understanding Swedish language, viable pregnancy in gestational age 14–24 weeks, intent to give birth and residing in the catchment area over the next couple of years.

Participants were recruited to the NorthPop study between May 2016 to May 2019. Informed consent was obtained from all participating women and their partners. Only singleton pregnancies from the NorthPop study were included in the analyses.

### Data collection

Data were collected using web questionnaires which were sent to the participating women at multiple times during and after the pregnancy. The first questionnaire was sent after inclusion (gestational age 17–25 weeks) and contained questions on socio-economic situation and medical history. A questionnaire at gestational age 32–34 weeks included questions about diet, physical activity, sedentary time and lifestyle during pregnancy. A questionnaire 4 months postpartum included questions about the woman’s health during pregnancy and the health of the newborn child. Questionnaire items used in the current study are listed in Supplemental Table 1. Additional data on parity, maternal body mass index (BMI; kg/m^2^) in early pregnancy, preeclampsia and pregnancy-induced hypertension diagnosis, self-rated health pre-pregnancy and during pregnancy, mode of delivery, gestational age, and blood loss during delivery and postpartum were obtained from the SPR, which is a national quality register [17, 18].

### Independent variables

*Maternal age* was defined as age at delivery. Information about the pregnant woman’s *country of birth* and *educational level* were retrieved from the first NorthPop web questionnaire. If data were missing (2.1%), corresponding data were retrieved from the SPR. *Country of birth* was divided into three groups; Sweden, other Nordic countries and other countries. Norway, Finland, Denmark and Iceland were included in the Nordic countries. *Educational level* was defined as the highest level of education achieved; elementary school, high school or university. *Parity* was divided into primiparity or multiparity. *BMI in early pregnancy* (kg/m^2^) was calculated from weight and height and women were stratified based on their BMI in early pregnancy according to the intervals used in the Institute of Medicine (IOM) guidelines for weight gain during pregnancy: Underweight < 18.5 kg/m^2^, Normal weight 18.5–24.9 kg/m^2^, Overweight 25.0–29.9 kg/m^2^ and Obese ≥30 kg/m^2^ [19]. *Self-rated health pre-pregnancy* had the following response alternatives: very poor, poor, neither good nor poor, good and very good.

### Dependent variables

Data on *GWG* was self-reported in the NorthPop web questionnaire and collected at 4 months postpartum. According to the IOM, underweight women should gain between 12.5–18 kg, those with normal weight 11.5–16 kg, those with overweight 7–11.5 kg and obese women should gain 5–9 kg during pregnancy [19]. Participants were classified into three categories based on the IOM guidelines: GWG lower than recommendations, within recommendations or exceeding the recommendations. *Birth weight* was categorized into low birth weight < 2500 g and high birth weight > 4000 g. Birth weight Z-scores were calculated based on a Swedish growth reference [20]. *Preeclampsia and pregnancy-induced hypertension* were categorized together and defined as having any of the following ICD10 diagnostic codes registered in the SRP; O14.0, O14.1, O14.1A, O14.1B, O14.1X, O14.2, O14.9, O13.9. *Mode of delivery* was defined as non-instrumental, vaginal instrumental, elective CS and emergency CS. *Gestational age at delivery* was divided into preterm (≤36 + 6), term (37 + 0–41 + 6) and post term (≥42 + 0). *Blood loss during delivery and postpartum* was defined as total amount of bleeding during delivery/postpartum in estimated milliliters and abnormal *delivery*/*postpartum hemorrhage* was defined as blood loss ≥500 ml.

### Level of physical activity and sedentary time during pregnancy

A questionnaire from the Swedish National Board of Health and Welfare (NBHW) based on validated categorical questions was used to estimate the level of PA during pregnancy [21]. PA was evaluated once during pregnancy and the NBHW questionnaire was included as a part of the NorthPop questionnaire sent out at gestational age 32–34 weeks. The questions, translated into English, are found in Supplemental Table 1. A total PA score was calculated by multiplying exercise time (scores for vigorous level PA) by two and adding the product to the score for every day (moderate level) PA time, providing an ordinal scale ranging from 3 to 19. The NBHW suggests that a total PA score of 11 or above represents the recommended level of PA [22]. Participants were divided into quartiles depending on their total activity score in one-way Analysis of variance (ANOVA) and Pearson’s Chi-square analysis. Sedentary time was also evaluated once during pregnancy in the NorthPop questionnaire sent out around gestational age 32–34 weeks and included a previously validated question about how many hours each day the participants spent sitting down, not including sleeping [23]. The response alternatives were: less than 1 h, 1–3 h, 4–6 h, 7–9 h, 10–12 h, 13–15 h and more than 15 h. The categories less than 1 h and 1–3 h and the categories 10–12 h, 13–15 h and more than 15 h were merged respectively in the ANOVA and Chi-square analysis to make the groups more equal in size.

### Statistical analyses

Possible differences between the categories were analyzed using T-test and ANOVA test for continuous variables and Pearson’s Chi-square test for categorical variables. Univariable and multivariable logistic regression analyses were used to assess effects on binary outcomes. PA was treated as a continuous variable from 3 to 19 in logistic regression analysis and linear regression analysis. Similarly, sedentary time was treated as a continuous variable from 1 to 7. Significance level was set to *p* < 0.05. Statistical analyses were done using SPSS version 26.

## Results

**Fig. 1:**
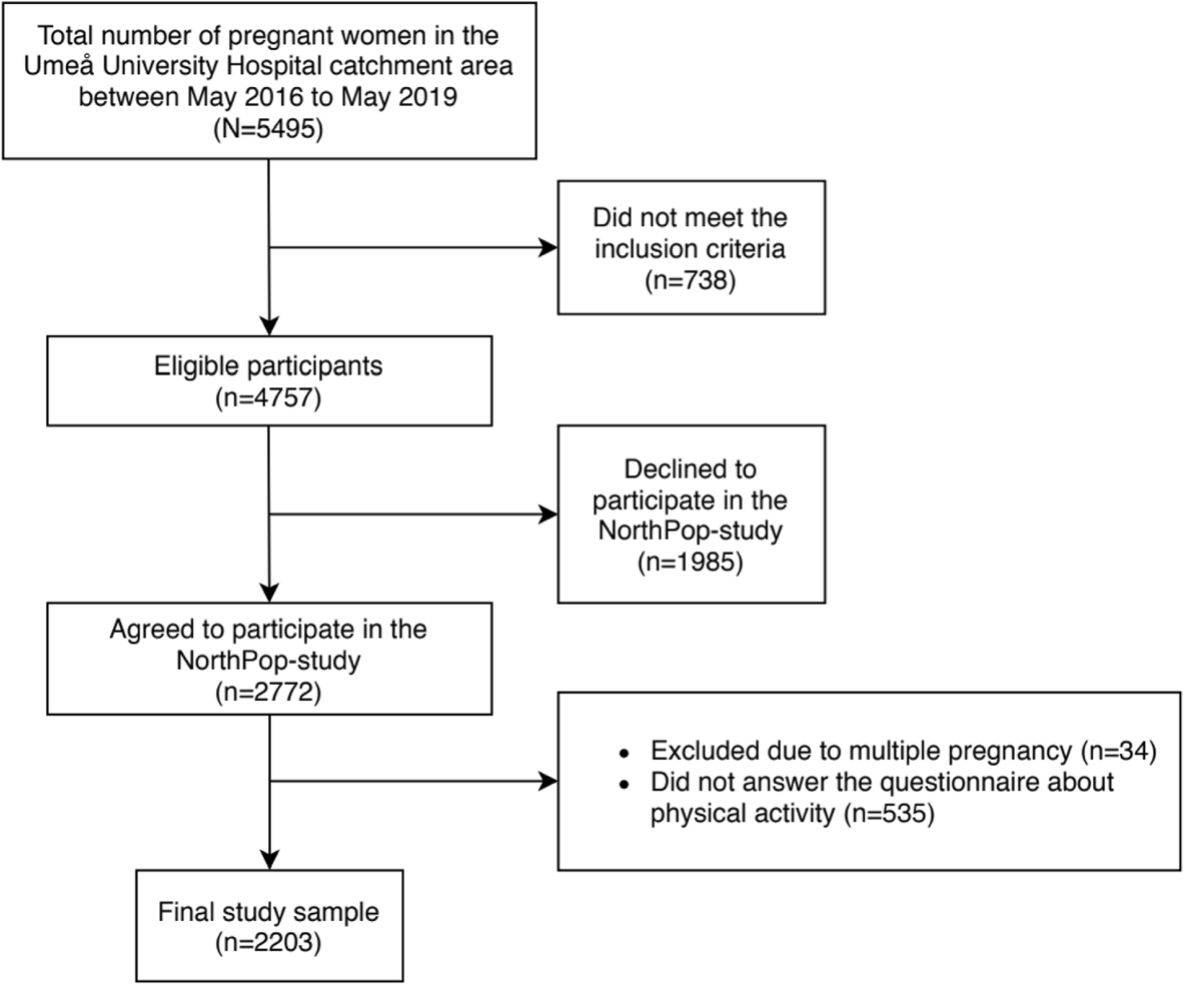
Flow chart presenting the study sample

From May 2016 to May 2019, 2772 women met the inclusion criteria for participating in the Northpop study and signed the informed consent. Only singleton pregnancies were included in this study. Of these, 2203 (80.5%) participants answered the questions regarding PA during pregnancy (Fig. 1). The questions were answered by the pregnant women in their 3rd trimester. Characteristics of non-responders (excluded participants) as compared to women included in the final study sample are presented in Supplementary Table 2.

### Physical activity

Of the included pregnant women, 27.3% reported ≥11 points on the total PA score, corresponding to the recommended level of PA. Distribution of participants according to total PA score are shown in Supplemental Figure 1. A higher level of reported PA during pregnancy was associated with a lower BMI in early pregnancy, higher educational level, Sweden as country of birth and a more favorable self-rated health pre-pregnancy. Among those participants who reached the recommendations, 62.6% were primiparous compared to the least active category where 36.4% were primiparous (Table 1).

**Table 1:** Background characteristics in relation to total PA score^a^ divided in quartiles

	n	1st quartile 3–5 p n (%)	2nd quartile 6–7 p n (%)	3rd quartile 8–10 p n (%)	4th quartile 11–19 p n (%)	***p***-value^**b**^
**Participants**	2203	531 (24.1)	459 (20.8)	611 (27.7)	602 (27.3)	
**Maternal age**	2203					
Mean; SD		31.0; 4.6	30.9; 4.5	30.7; 4.3	30.9; 4.2	0.791
**Country of birth**	2197					
Sweden		459 (86.9)	419 (91.7)	562 (92.0)	557 (92.7)	**< 0.001**
Other nordic countries		2 (0.4)	6 (1.3)	8 (1.3)	9 (1.5)	
Other countries^c^		67 (12.7)	32 (7.0)	41 (6.7)	35 (5.8)	
**Educational level**	2197					
Elementary school		28 (5.3)	9 (2.0)	20 (3.3)	3 (0.5)	**< 0.001**
High school		162 (30.6)	142 (31.1)	156 (25.5)	140 (23.3)	
University		339 (64.1)	306 (67.0)	435 (71.2)	457 (76.2)	
**BMI in early pregnancy**^**d**^	2132					
Mean; SD		25.3; 4.6	25.2; 4.8	24.4; 4.1	23.8; 3.8	**< 0.001**
< 18.5		8 (1.6)	14 (3.1)	8 (1.4)	12 (2.1)	**< 0.001**
18.5–24.99		274 (53.6)	251 (56.2)	382 (64.4)	403 (69.1)	
25–29.99		148 (29.0)	110 (24.6)	142 (24.0)	126 (21.6)	
≥ 30		81 (15.9)	72 (16.1)	59 (10.0)	42 (7.2)	
**Parity**	2192					
Primiparous		192 (36.4)	210 (46.0)	295 (48.4)	374 (62.6)	**< 0.001**
Multiparous		336 (63.6)	247 (54.0)	315 (51.6)	223 (37.4)	
**Self-rated health pre-pregnancy**	1947					
Very poor/poor		14 (3.0)	12 (2.9)	10 (1.8)	6 (1.1)	**< 0.001**
Neither good nor poor		55 (12.0)	31 (7.6)	38 (7.0)	23 (4.3)	
Good/very good		391 (85.0)	364 (89.4)	496 (91.2)	507 (94.6)	

^a^Calculated by multiplying scores for vigorous level PA by two and adding the product to the score for moderate level PA time. Higher score represents higher level of PA

^b^ANOVA test for continuous variables and Chi-square test for categorical variables

^c^All other countries

^d^Body Mass Index. (kg/m^2^)

**Fig. 2:**
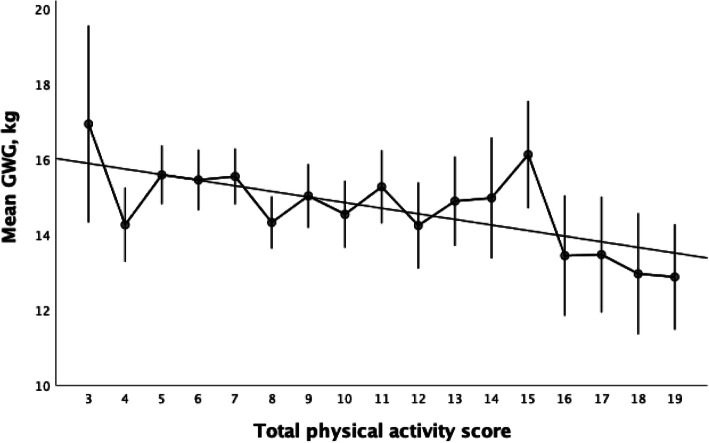
Correlation between total PA score and mean GWG in each category, presented with 95% CI and trendline

Total PA score was weakly correlated with sedentary time (r − 0.077, *p* < 0.001). There was a fairly weak negative association between total PA score and GWG which remained significant (r − 0.087, *p* < 0.001) in a multivariable regression analysis, adjusting for BMI in early pregnancy, country of birth, educational level, parity and self-rated health pre-pregnancy (Fig. 2).

In total 48.7% of the pregnant women exceeded the IOM recommended GWG. A higher total PA score was associated with a lower risk of exceeding the IOM recommended GWG (OR 0.94, 95% CI 0.91–0.96) and a lower risk of emergency CS (OR 0.93, 95% CI 0.89–0.98). A higher level of total PA was also associated with a lower risk of reporting a poor or very poor self-rated health during pregnancy (OR 0.84, 95% CI 0.77–0.91). These results remained significant after adjusting for BMI in early pregnancy, country of birth, educational level, parity and self-rated health pre-pregnancy.

There was a weak negative correlation between total PA score and birth weight z-score in linear regression analysis (r − 0.083, *p* < 0.001), although not significant in multivariable analysis. There was also a negative association between PA and birth weight > 4000 g (OR 0.97, 95% CI 0.94–0.998), but the result did not remain significant in multivariable analysis. There was no significant association between total PA score and risk of pregnancy-induced hypertension and/or preeclampsia diagnosis, gestational age and blood loss during delivery and postpartum (Table 2).

**Table 2:** Maternal and fetal/child outcomes in relation to total PA score^a^ divided in quartiles

	1st quartile 3–5 p n (%)	2nd quartile 6–7 p n (%)	3rd quartile 8–10 p n (%)	4th quartile 11–19 p n (%)	***p***-value^**b**^
**Gestational age (days)**					
Mean; SD	279.0; 10.2	278.8; 9.7	279.1; 9.9	279.3; 12.0	0.913
Preterm^c^	21 (4.0)	13 (2.8)	18 (2.9)	29 (4.8)	0.072
Term^c^	469 (88.8)	426 (93.2)	555 (90.8)	524 (87.5)	
Post term^c^	38 (7.2)	18 (3.9)	38 (6.2)	46 (7.7)	
**Gestational weight gain**					
Mean; SD	15.3; 6.6	15.5; 5.6	14.6; 5.6	14.5; 5.3	**0.021**
Lower than recommendations^d^	64 (14.3)	60 (15.1)	98 (18.3)	114 (21.3)	**< 0.001**
Within recommendations^d^	134 (29.9)	113 (28.5)	192 (35.9)	208 (38.8)	
Exceeding recommendations^d^	250 (55.8)	224 (56.4)	245 (45.8)	214 (39.9)	
**Birth weight (g**^**e**^**)**					
Mean; SD	3603.9; 492.5	3589.9; 502.1	3536.8; 497.0	3525.5; 533.1	**0.022**
< 2500	5 (0.9)	8 (1.7)	11 (1.8)	15 (2.5)	0.077
2500–4000	414 (78.0)	358 (78.0)	507 (83.0)	479 (79.6)	
> 4000	112 (21.1)	93 (20.3)	93 (15.2)	108 (17.9)	
**Mode of delivery**					
Non-instrumental	404 (76.5)	375 (82.1)	501 (82.0)	492 (82.1)	**0.005**
Vag. Instr.^f^	17 (3.2)	19 (4.2)	20 (3.3)	35 (5.8)	
Elective CS^g^	47 (8.9)	29 (6.3)	39 (6.4)	25 (4.2)	
Emergency CS^g^	60 (11.4)	34 (7.4)	51 (8.3)	47 (7.8)	
**Blood loss during delivery/postpartum (ml**^**h**^**)**^**i**^					
Mean; SD	517.2; 362.0	534.4; 392.3	533.2; 408.1	515.8; 379.7	0.778
≥ 500	129 (31.3)	119 (31.8)	175 (35.0)	168 (34.2)	0.674
**Preeclampsia/ Pregnancy-induced hypertension**	15 (2.8)	16 (3.5)	29 (4.8)	20 (3.3)	0.348
**Self-rated health during pregnancy**					
Very poor/poor	36 (10.6)	19 (6.4)	26 (6.7)	10 (2.4)	**< 0.001**
Neither good nor poor	53 (15.6)	49 (16.6)	40 (10.3)	33 (7.8)	
Good/Very good	250 (73.7)	228 (77.0)	322 (83.0)	378 (89.8)	

^a^Calculated by multiplying scores for vigorous level PA by two and adding the product to the score for moderate level PA time. Higher score represents higher level of PA

^b^ANOVA test for continuous variables and Chi-square test for categorical variables

^c^Preterm (≤36 + 6), term (37 + 0–41 + 6) and post term (≥42 + 0)

^d^According to the IOM, underweight women should gain between 12.5–18 kg, those with normal weight 11.5–16 kg, those with overweight 7–11.5 kg and obese women should gain 5–9 kg during pregnancy

^e^Grams (g)

^f^Vaginal instrumental. Including vacuum extraction or forceps

^g^Caesarean section (CS)

^h^Milliliters (ml)

^i^Only vaginal non-instrumental deliveries included

### Sedentary time

The largest proportion of the participating women (34.6%) reported between 4 and 6 h of sedentary time per day. Distribution of participants according to sedentary time per day are shown in Supplemental Figure 2. Participants in the categories with higher sedentary time demonstrated significantly higher maternal age, higher educational level, and larger proportions were primiparous and reported Sweden as country of birth compared to those with lower sedentary time (Table 3).

**Table 3:** Background characteristics in relation to amount of sedentary time per day in categories

	n	< 3 h n (%)	4-6 h n (%)	7-9 h n (%)	≥10 h n (%)	***p***-value^**a**^
**Participants**	2201	295 (13.4)	761 (34.6)	642 (29.2)	503 (22.9)	
**Maternal age**	2201					
Mean; SD		30.7; 4.3	30.5; 4.4	31.0; 4.3	31.3; 4.4	**0.013**
**Country of birth**	2195					
Sweden		251 (85.7)	687 (90.5)	589 (91.9)	496 (93.4)	**0.005**
Nordic countries		3 (1.0)	7 (0.9)	9 (1.4)	6 (1.2)	
Other countries^b^		39 (13.3)	65 (8.6)	43 (6.7)	27 (5.4)	
**Educational level**	2195					
Elementary school		14 (4.8)	28 (3.7)	8 (1.3)	10 (2.0)	**< 0.001**
High school		134 (45.7)	240 (31.6)	145 (22.7)	80 (15.9)	
University		145 (49.5)	492 (64.7)	487 (76.1)	412 (82.1)	
**BMI in early pregnancy**^**c**^	213					
Mean; SD		24.7; 4.3	24.5; 4.2	24.5; 4.4	24.8; 4.5	0.790
< 18.5		5 (1.8)	15 (2.0)	13 (2.1)	9 (1.9)	
18.5–24.99		172 (60.8)	455 (61.2)	388 (62.7)	293 (60.4)	
25–29.99		73 (25.8)	195 (26.2)	138 (22.3)	120 (24.7)	
≥ 30		33 (11.7)	78 (10.5)	80 (12.9)	63 (13.0)	
**Parity**	2190					
Primiparous		100 (34.1)	359 (47.5)	331 (51.6)	281 (56.2)	**< 0.001**
Multiparous		193 (65.9)	397 (52.5)	310 (48.4)	219 (43.8)	
**Self-rated health pre-pregnancy**	1945					
Very poor/poor		4 (1.5)	17 (2.5)	9 (1.6)	12 (2.7)	0.292
Neither good nor poor		16 (6.1)	45 (6.7)	54 (9.6)	32 (7.1)	
Good/Very good		244 (92.4)	608 (90.7)	497 (88.8)	407 (90.2)	

^a^ANOVA test for continuous variables and Chi-square test for categorical variables

^b^All other countries

^c^Body Mass Index (kg/m^2^)

Higher sedentary time was weakly associated with increased blood loss during delivery/postpartum in linear regression analysis (r 0.06, p 0.02) and increased risk of blood loss ≥500 ml during delivery/postpartum (OR 1.11, 95% CI 1.02–1.21). This result remained significant after adjusting for maternal age, country of birth, educational level, BMI in early pregnancy and parity. There was a borderline significant association between higher sedentary time and risk of pregnancy-induced hypertension and/or preeclampsia (OR 1.21, 95% CI 1.00–1.50). The prevalence of pregnancy-induced hypertension and/or preeclampsia was significantly higher among those with sedentary time ≥ 7 h compared to those with sedentary time < 7 h (4.5% vs 2.8%; OR 1.65, 95% CI 1.03–2.62), but after adjusting for BMI and parity this result was no longer significant. No association was found between sedentary time and the risk of answering “poor or very poor” on the question about self-rated health during pregnancy. However, there was a significant association between sedentary time and answer “neither good nor poor” instead of “good or very good” on the question related to self-rated health (OR 1.32, 95% CI 1.15–1.52). The OR slightly increased after adjusting for maternal age, country of birth, educational level, BMI in early pregnancy, parity and PA during pregnancy (OR 1.37, 95% CI 1.19–1.58). Sedentary time was not significantly associated with mode of delivery, gestational age, GWG or birth weight of the child (Table 4).

**Table 4:** Maternal and fetal/child outcomes in relation to sedentary time per day in categories

	< 3 h n (%)	4-6 h n (%)	7-9 h n (%)	≥10 h n (%)	***p***-value^**a**^
**Gestational age**					
Mean; SD	279.4; 10.4	278.8; 10.8	278.9; 10.5	279.7; 10.3	0.412
Preterm^b^	10 (3.4)	33 (4.4)	21 (3.3)	17 (3.4)	0.094
Term^b^	254 (86.4)	677 (89.4)	589 (91.7)	452 (90.4)	
Post term^b^	30 (10.2)	47 (6.2)	32 (5.0)	31 (6.2)	
**Gestational weight gain**					
Mean; SD	14.2; 5.8	15.0; 5.8	15.3; 5.8	14.8; 5.5	0.102
Lower than recommendations^c^	50 (20.2)	117 (17.7)	91 (16.2)	78 (17.5)	0.837
Within recommendations^c^	86 (34.8)	217 (32.8)	193 (34.4)	151 (33.9)	
Exceeding recommendations^c^	111 (44.9)	327 (49.5)	277 (49.4)	217 (48.7)	
**Birth weight (g**^**d**^**)**					
Mean; SD	3558.1; 504.2	3554.1; 515.6	3551.4; 493.3	3585.0; 518.2	0.612
< 2500	1 (0.3)	19 (2.5)	8 (1.2)	11 (2.2)	0.073
2500–4500	238 (80.7)	604 (79.4)	528 (82.2)	386 (76.7)	
> 4000	56 (19.0)	138 (18.1)	106 (16.5)	106 (21.1)	
**Mode of delivery**					
Non-instrumental	242 (82.3)	620 (81.9)	520 (81.0)	389 (77.8)	0.272
Vag. Instr.^e^	11 (3.7)	34 (4.5)	20 (3.1)	26 (5.2)	
Elective CS^f^	12 (4.1)	48 (6.3)	46 (7.2)	33 (6.6)	
Emergency CS^f^	29 (9.9)	55 (7.3)	56 (8.7)	52 (10.4)	
**Blood loss during delivery/postpartum (ml**^**g**^**)**^**h**^					
Mean; SD	483.1; 339.5	464.1; 357.0	502.0; 399.4	538.6; 369.3	**0.013**
≥ 500	78 (32.4)	184 (29.7)	173 (33.3)	156 (40.1)	**0.008**
**Preeclampsia/ Pregnancy-induced hypertension**	7 (2.4)	22 (2.9)	31 (4.8)	20 (4.0)	0.156
**Self-rated health during pregnancy**					
Very poor/poor	11 (5.8)	32 (6.6)	28 (6.5)	20 (5.9)	**0.037**
Neither good nor poor	14 (7.3)	52 (10.7)	51 (11.9)	58 (17.2)	
Good/Very good	166 (86.9)	402 (82.7)	349 (81.5)	260 (76.9)	

^a^ANOVA test for continuous variables and Chi-square test for categorical variables

^b^Preterm (≤36 + 6), term (37 + 0–41 + 6) and post term (≥42 + 0)

^c^According to the IOM, underweight women should gain between 12.5–18 kg, those with normal weight 11.5–16 kg, those with overweight 7–11.5 kg and obese women should gain 5–9 kg during pregnancy

^d^Grams (g)

^e^Vaginal instrumental. Including vacuum extraction or forceps

^f^Caesarean section (CS)

^g^Milliliters (ml)

^h^Only vaginal non-instrumental deliveries included

## Discussion

The main findings in this study showed that PA during pregnancy was associated with lower mean GWG, reduced risk of exceeding the IOM recommended GWG, and a reduced risk of emergency CS. Longer sedentary time during pregnancy was also found to be weakly associated with an increased blood loss during delivery/postpartum. Both PA and sedentary time were significantly associated with reported self-rated health during pregnancy.

A lower proportion (27.3%) of the participants reached the recommended level of PA during pregnancy until gestational age 32–34 weeks as compared to a previous study that investigated the pre-pregnant PA level and PA until gestational age 10 weeks in the Swedish pregnant population, in which 47% reported that they reached the recommended level of PA [16]. A possible explanation for the difference between the outcomes of the studies is that pregnant women tend to decrease their PA later in pregnancy [24]. Different methods for estimating the participants’ level of PA may also have affected the results. The proportion of pregnant women who reached the recommendations in the 3rd trimester was at similar levels compared to previous studies in other countries reporting 28 and 27% reaching ≥150 min of moderate PA/week [7, 25]. Higher educational level and having no other children at home have been associated to higher level of PA [26]. In our study, pregnant women in the categories reporting a higher level of PA during pregnancy also presented a more favorable self-rated health pre-pregnancy, lower BMI in early pregnancy and larger proportions born in Sweden.

Previous studies have found PA during pregnancy to be inversely associated with excessive GWG [5, 27, 28]. Our result is consistent with these studies. There was a lower proportion of pregnant women exceeding the IOM recommendations in the categories with at least eight points on the total PA score compared to the categories with less than eight points, indicating that moderate PA representing less time than the recommended 150 min each week may have a positive effect if it is combined with at least a few minutes of vigorous PA/week.

The highest proportion of emergency CS (11.4%) was seen in the category with the lowest PA level in our study, as compared to 7.4–8.3% in the other categories. This result is in line with some other observational studies examining PA and the risk of emergency CS [29, 30]. In a recent meta-analysis, exercise during pregnancy was associated with a reduction of instrumental deliveries but not associated with the risk of delivery by CS [31]. The difference in results may be due to the fact that this meta-analysis included all types of CS while we included only emergency CS. A possible explanation for the association between PA and emergency CS is that pregnant women with a lower level of PA had a higher risk of exceeding the IOM recommended GWG, and a GWG above the recommendations is associated with increased risk of emergency CS [32].

A novel finding in our study was that sedentary time was associated with a non-favorable self-rated health during pregnancy, independently of the pregnant woman’s level of PA. To our knowledge, this association has not been demonstrated previously in a pregnant population and could be of importance when giving advice to improve the pregnant woman’s self-rated health during pregnancy. More studies are thus needed to confirm this association. We also found that longer sedentary time was associated with a slightly increased blood loss during delivery/postpartum. An increase in postpartum hemorrhage might be associated with obesity [33], but women with more sedentary time did not present a higher BMI in our study. However, the association we found in or study was weak and other explanations for this association is possible.

Reported median sedentary time in our study was 7–9 h, which is similar to an observational study among Chinese, Malay and Indian women which reported a median sitting time of 9 h per day [34]. The self-reported sedentary time in our study was also similar to studies using objective methods which have reported mean sedentary time during pregnancy of 9.3 and 7.1 h per day respectively [35, 36]. Participants in the categories with higher sedentary time demonstrated higher maternal age, higher educational level and larger proportions were primiparous and born in Sweden. Taken the results from the PA analysis into account, this indicates that the participants who reported a higher educational level and were primiparous more often reached the recommended level of PA, while still spending a significant amount of time sedentary. This is in line with earlier studies that have shown that even if an individual reaches the recommended level of PA, the same individual may still spend many hours each day being inactive [7]. A possible explanation is that women with more than one child may have less time to perform PA, but still spend more time on daily activities like housework and playing with the children, and thus spending less time sedentary [26].

Higher incidence of preeclampsia and/or pregnancy-induced hypertension was observed among women who reported higher sedentary time, although this association did not remain significant when controlling for BMI and parity. However, the loss of significance when including BMI in the model could indicate a pathway for the effect of sedentary time on hypertension via an increased BMI. PA interventions have previously been shown to reduce the risk of hypertension disorders during pregnancy [4, 37]. The effect of sedentary time on these disorders are not well investigated, and two previous studies present no significant association [38, 39]. The disparity in results may depend on differences in the methods for estimating the participants sedentary behavior, and more studies are needed to evaluate the effect of sedentary time on pregnancy-induced hypertension and preeclampsia.

### Methodological considerations

This study has some limitations: PA level and sedentary time were self-reported in the questionnaires from the NorthPop study, which may contribute to an overestimation or underestimation of PA time compared to accelerometer data [40]. The questions about level of PA and sedentary time were also asked at a single timepoint in the third trimester, so we cannot evaluate any changes in level of PA and sedentary time during the course of pregnancy. Another limitation related to self-reported data is that we were not able to analyze detailed patterns of PA that might have affected our outcomes. GWG was also self-reported and an underestimation of the number of women exceeding the IOM recommended GWG is possible. Blood loss during delivery/postpartum was also estimated and not measured precisely. The categorical questions about PA are validated among Swedish adults in general, but not specifically in the pregnant population. However, the questions have shown strong correlation with objective accelerometer measured PA [21]. Pregnant women in Sweden are recommended to engage in PA of total 30 min a day, on most days of the week [3]. Through our questionnaire we were only able to evaluate the total amount of PA in a week, and therefore women were considered to have reached the recommendations if they achieved ≥150 min of moderate-vigorous PA each week, although we cannot be sure that the time was spread on multiple days. As with all research on PA, it might also be a problem determining the direction of causality. PA can be effective for preventing certain symptoms or diseases, but women who have these symptoms or diseases in advance might be afraid that PA during pregnancy will affect the fetus negatively and will therefore avoid PA. Participants who were excluded due to not responding to the questionnaire on PA, reported a lower education level and were more often of non-Swedish origin (Supplemental Table 2).

Strengths of this study is that both the NorthPop study and the SPR have a high coverage of the population in the catchment area (64 and 85%, respectively) and most of the variables in the SPR are transferred electronically from medical records [17, 18]. Several of the variables in our study were also available both in the NorthPop and in the SPR databases and could therefore be validated against each other.

## Conclusions

Despite advice regarding PA being given to the pregnant women, only a low proportion of the participants in our study reported that they reached the recommended level of PA. An alarmingly large proportion of the participants also gained more weight than recommended during pregnancy (49%). Encouraging pregnant women to increase their PA and decrease their sedentary time may be important to reduce the risk of excessive GWG and improve the health of pregnant women and their offspring. However, further studies are needed to evaluate the impact of different levels of sedentary time on pregnancy outcomes and possibly demonstrate evidence to make recommendations for limiting sedentary time during pregnancy.

## Supplementary Information

**Additional file 1: Supplemental Table 1.** Questionnaire questions used in this study^a^.

**Additional file 2: Supplemental Figure 1.** Distribution of participants according to total physical activity score.

**Additional file 3: Supplemental Figure 2.** Distribution of participants according to sedentary time per day.

**Additional file 4: Supplemental Table 2.** Characteristics of excluded participants as compared to participants included in the final study sample.

## Data Availability

The datasets analyzed during the current study are not publicly available due to regulations on sensitive personal data but are available from the corresponding author on reasonable request.

## Abbreviations

BMI: Body Mass Index
CS: Caesarean section
GWG: Gestational weight gain
IOM: Institute of Medicine
PA: Physical activity
NBHW: National Board of Health and Welfare
SPR: Swedish Pregnancy Register
WHO: World Health Organization
