# Supplementary material for: Physical activity and sedentary time during pregnancy and associations with maternal and fetal health outcomes: an epidemiological study
Source: BMC Pregnancy Childbirth. 2021 Feb 27;21:166. doi: 10.1186/s12884-021-03627-6 (PMC7913456; doi:10.1186/s12884-021-03627-6)
Supplement: Supplementary file 1 — Additional file 1: Supplemental Table 1. Questionnaire questions used in this studya. [file 12884_2021_3627_MOESM1_ESM.docx]

**Supplemental Table 1.** Questionnaire questions used in this study.^a^

| Country of birth. Where were you born?^b^ |
| --- |
| What is the highest level of education you have achieved?^b^   - Less than 9-year elementary school - 9-year elementary school - High school - University |
| During a regular week, how much time do you spend on daily exercise like walking, biking, snow shoveling or mowing the lawn? This includes walking or taking the bike to work.^c^ |
| - 0 minutes - Less than 30 minutes - 30-60 minutes - 1-1,5 hours - 1,5-2,5 hours - 2,5-5 hours - More than 5 hours |
| During a regular week, how much time do you spend on physical activity that makes you short-winded, like running, aerobic classes, spinning, ball games etc.? (Summarize all the time, at least 10 minutes at a time).^c^ |
| - Do not exercise (0 min) - Less than 30 minutes - 30-60 minutes - 60-90 minutes - 90-120 minutes - More than 120 minutes |
| During a regular day, how much time do you spend sitting down, not including sleeping? For example, sitting at a desk or dining table, in front of a computer, TV (incl. Video games), sitting in a car or a bus. Count all time, both at work and at leisure time.^c^ |
| - Less than 1 hour |
| - 1-3 hours |
| - 4-6 hours |
| - 7-9 hours - 10-12 hours - 13-15 hours - More than 15 hours |
|  |
| Gestational weight gain. How many kilograms did you gain in total during pregnancy? Enter the weight gain in kilograms, integers numbers.^d^ |

^a^ The questions are originally written in Swedish but have for the purpose of this publication been translated into English.

^b^ Included in the first web questionnaire sent out around gestational age 17-25 weeks.

^c^ Included in the web questionnaire sent out around gestational age 32-34 weeks.

^d^ Included in the web questionnaire sent out four months postpartum.
