# Supplementary figures and images for: Physical activity and sedentary time during pregnancy and associations with maternal and fetal health outcomes: an epidemiological study
Source: BMC Pregnancy Childbirth. 2021 Feb 27;21:166. doi: 10.1186/s12884-021-03627-6 (PMC7913456; doi:10.1186/s12884-021-03627-6)

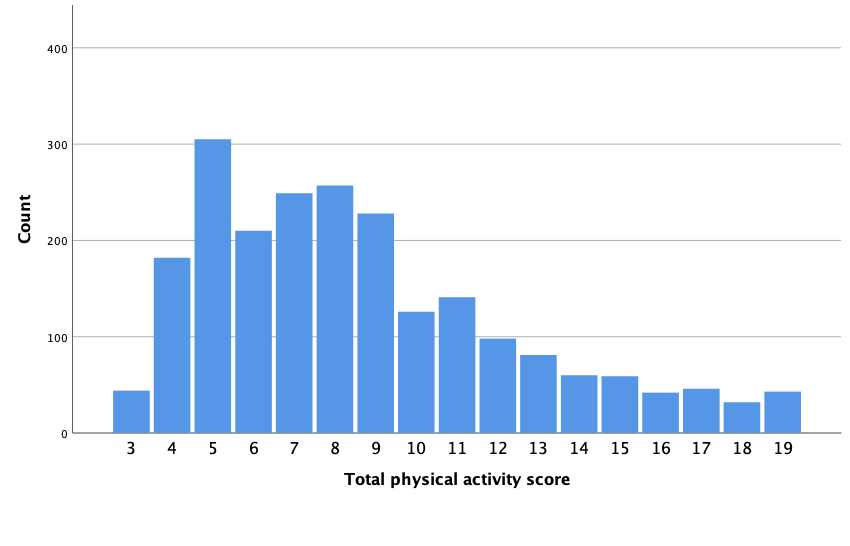

Supplement: Supplementary file 2 — Additional file 2: Supplemental Figure 1. Distribution of participants according to total physical activity score. [file 12884_2021_3627_MOESM2_ESM.docx]

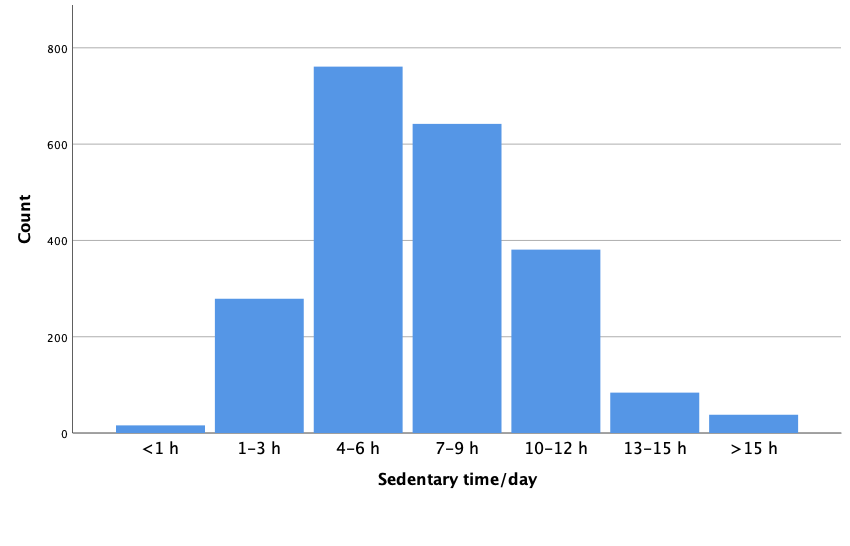

Supplement: Supplementary file 3 — Additional file 3: Supplemental Figure 2. Distribution of participants according to sedentary time per day. [file 12884_2021_3627_MOESM3_ESM.docx]
