# Supplementary material for: Physical activity and sedentary time during pregnancy and associations with maternal and fetal health outcomes: an epidemiological study
Source: BMC Pregnancy Childbirth. 2021 Feb 27;21:166. doi: 10.1186/s12884-021-03627-6 (PMC7913456; doi:10.1186/s12884-021-03627-6)
Supplement: Supplementary file 4 — Additional file 4: Supplemental Table 2. Characteristics of excluded participants as compared to participants included in the final study sample. [file 12884_2021_3627_MOESM4_ESM.docx]

**Supplemental Table 2.** Characteristics of excluded participants as compared to participants included in the final study sample

|  | n | Excluded^a^  n (%) | Final study sample  n (%) | | p-value^b^ |  |
| --- | --- | --- | --- | --- | --- | --- |
| Participants | 2738 | 535 (19.5) | 2203 (80.5) |  |  |  |
|  |  |  |  |  |  |  |
| **Maternal age** | 2738 |  |  |  |  |  |
| Mean; SD |  | 30.5; 4.8 | 30.9; 4.4 |  | 0.067 |  |
|  |  |  |  |  |  |  |
| **Country of birth** | 2696 |  |  |  |  |  |
| Sweden |  | 411 (82.4) | 1997 (90.9) |  | **<0.001** |  |
| Other Nordic countries | | 3 (0.6) | 25 (1.1) |  |  |  |
| Other countries^c^ | | 85 (17.0) | 175 (8.0) |  |  |  |
|  |  |  |  |  |  |  |
| **Educational level** | 2683 |  |  |  |  |  |
| Elementary school | | 38 (7.8) | 60 (2.7) |  | **<0.001** |  |
| High school |  | 143 (29.4) | 600 (27.3) |  |  |  |
| University |  | 305 (62.8) | 1537 (70.0) |  |  |  |
|  |  |  |  |  |  |  |
| **BMI in early pregnancy**^d^ | 2645 |  |  |  |  |  |
| Mean; SD |  | 24.9; 4.7 | 24.6; 4.3 |  | 0.203 |  |
|  |  |  |  |  |  |  |
| **Parity** | 2721 |  |  |  |  |  |
| Primiparous |  | 238 (45.0) | 1071 (48.9) |  | 0.110 |  |
| Multiparous |  | 291 (55.0) | 1121 (51.1) |  |  |  |
|  |  |  |  |  |  |  |
| **Self-rated health pre-pregnancy** | 2397 |  |  |  |  |  |
| Very poor/poor | | 9 (2.0) | 42 (2.2) |  | 0.444 |  |
| Neither good nor poor | | 42 (9.3) | 147 (7.6) |  |  |  |
| Good/very good | | 399 (88.7) | 1758 (90.3) |  |  |  |

^a^ Women excluded due to not answering the questionnaire about PA.

^b^ T-test for continuous variables and Chi-square test for categorical variables.

^c^ All other countries.

^d^ Body Mass Index. (kg/m^2^)
